# Pulmonary Epithelioid Hemangioendothelioma in a Patient with Crohn's Disease

**DOI:** 10.1155/2015/491960

**Published:** 2015-02-28

**Authors:** Nanda Ramchandar, Henry A. Wojtczak

**Affiliations:** Naval Medical Center San Diego, Department of Pediatrics, 34800 Bob Wilson Drive, San Diego, CA 92134, USA

## Abstract

Pulmonary epithelioid hemangioendothelioma (PEH) is a rare neoplasm, largely unresponsive to chemotherapeutic medications, and with varied prognosis. Imaging on computerized tomography may demonstrate perivascular nodules, but diagnosis is ultimately made on biopsy with immunohistochemical analysis. Here we describe a case of PEH in a 14-year-old male with Crohn's disease, which, to our knowledge, has not previously been described in the literature.

## 1. Introduction

Originally described as an intravascular bronchoalveolar tumor by Dail and Liebow in 1975, pulmonary epithelioid hemangioendothelioma (PEH) is a rare vascular neoplasm that may arise as a primary tumor either in the lung or in the pleura [1–4]. The natural history and clinical course of PEH are poorly understood, and malignant potential ranges from benign hemangioma to malignant angiosarcoma. Diagnosis is made on histology, requiring immunohistochemical staining for markers specific to vascular endothelium. Response to chemotherapy is minimal at best with prognosis varying from complete spontaneous regression to rapid onset of end stage disease. To our knowledge, there is only one case in the literature of a hepatic epithelioid hemangioendothelioma and there are no cases of PEH described in the setting of inflammatory bowel disease [5]. We report a 14-year-old male with Crohn's disease (CD) and pulmonary epithelioid hemangioendothelioma.

## 2. Case Report

**Figure 1:**
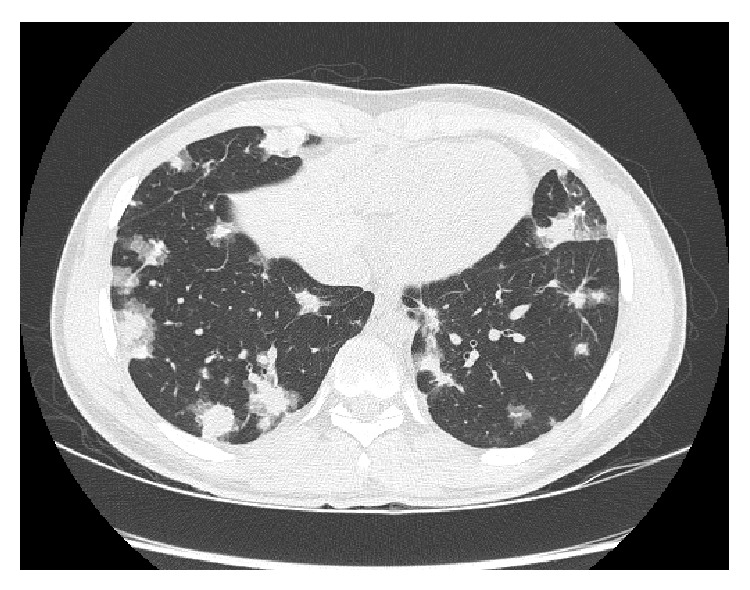
Chest computerized tomography scan. Multiple irregular ground glass nodules 2-3 cm in diameter, mild thickening of airway walls, no effusion, and reactive lymphadenopathy in the perihilar regions bilaterally.

A 14-year-old male diagnosed with Crohn's disease in August of 2012 is referred to a pulmonologist for multiple pulmonary nodules found incidentally on abdominal computerized tomography during evaluation of his Crohn's disease. Initially, there was no complaint of respiratory symptoms, but over time, he developed a dry cough and back pain. Patient was well-developed and well-nourished on physical exam with symmetric chest. Lung exam was without crackles or wheeze, but with mildly prolonged expiratory phase. There was no hepatosplenomegaly, tenderness, or distension on the abdominal exam. Chest radiograph findings included multiple pulmonary nodules, and chest CT showed irregular ground glass nodules 2-3 cm in diameter, mild thickening of airway walls, no effusion, and reactive lymphadenopathy in the perihilar regions bilaterally (Figure 1).

Spirometry showed forced vital capacity (FVC) of 2.78 (78%), forced expiratory volume in 1 second (FEV1) of 2.10 (68%), FEV1/FVC of 76%, and forced expiratory flow 25–75% (FEF 25–75%) of 46%. Extensive pulmonary workup for pulmonary nodules including bronchoscopy, bronchoalveolar lavage (BAL), and thoracoscopic lung biopsy was completed. Results were negative for infectious, rheumatologic, or malignant processes. Of note, there was grossly bloody BAL fluid and numerous red blood cells. Lung biopsy revealed multifocal intra-alveolar collections of hemosiderin-laden macrophages with focal hemorrhage, scattered pulmonary artery branches showing collapse with luminal loss and mural calcification, and no vasculitis. Surrounding the degenerated arteries were areas of fibrosis with peripheral aerated alveolar spaces present. Final pathology reported arteriopathy with hemosiderosis, hemorrhage, and fibrosis. Despite regular infliximab infusions for his CD, pulmonary symptoms did not resolve. Patient was started on 3-week course of prednisone, but again to no avail. Patient underwent a second bronchoscopy and repeat thoracoscopic lung biopsy. Analysis of biopsies showed nodular cellular infiltrate intermixed with hemosiderin laden macrophages and plugs of fibrin. Atypical cells were epithelioid in appearance, consistent with PEH. Immunohistochemical staining confirmed endothelial lineage.

## 3. Discussion

PEH was originally described in 1975 as an aggressive form of bronchoalveolar cell carcinoma with invasion into surrounding vasculature [1, 2, 6]. Subsequent immunochemical analysis and electron microscopy revealed the endothelial nature of this neoplasm, and the current name of PEH was coined by Weiss et al. in 1982 [3, 4, 6]. Epithelioid hemangioendothelioma is not specific to the lung, arising as a primary lesion more commonly in the liver as well as bone and other soft tissues. It is more often diagnosed in women and in younger patients, with a median age of 35 years [6–8]. Imaging is not sufficient for diagnosis, but lesions often appear as either unilateral or bilateral perivascular nodules generally <2 cm in diameter [6, 9]. Histological characteristics include nodules with hypocellular sclerotic or necrotic center. Immunochemical staining for vimentin, erythroblast transformation-specific related gene (ERG), cluster of differentiation 31, cluster of differentiation 34, and factor VIII or friend leukemia integration 1 (FLI-1) transcription factor confirms diagnosis [4, 6, 10, 11]. Additionally, use of fluorescence in situ hybridization (FISH) or polymerase chain reaction (PCR) to detect CAMTA1-WWTR1 and YAP1-TFE3 rearrangements can be used as an adjunct to immunohistochemical analysis, tools that are useful in distinguishing epithelioid hemangioendothelioma from other epithelioid vascular neoplasms [10–13].

Prognosis is difficult to forecast, ranging from complete resolution without intervention to rapid progression and death. Poor prognostic indicators include low weight, anemia, pulmonary symptoms, pleural hemorrhagic effusions, and hemoptysis [14, 15]. In patients with pleural effusion or hemoptysis, the median survival is less than 1 year [4, 10, 14]. Conversely, in patient with asymptomatic pulmonary nodules, average survival time is 15 years [8]. There is currently no gold standard for therapy given the relative rarity of this condition. Chemotherapeutic measures have been attempted with varying degrees of success, but PEH is often unresponsive to treatment [16]. Such agents used in the treatment of PEH include vincristine, cisplatin, 5-fluorouracil, mitomycin, cyclophosphamide, ifosfamide, and etoposide [16]. Radiation therapy has been attempted as well. In asymptomatic patients, careful observation can result in spontaneous regression [14, 15]. With regard to our patient, he developed hemoptysis 3 months after his repeat lung biopsy. Imaging did not demonstrate progression of nodules, and spirometry remained stable, so close observation was chosen as a reasonable treatment modality.

CD, an inflammatory bowel disease (IBD) characterized by chronic, granulomatous inflammation of the intestines, is associated with many concomitant respiratory ailments, including pulmonary nodules and bronchiectasis. The pulmonary nodules associated with CD are sterile necrobiotic lesions that are generally responsive to corticosteroid therapy [17–20]. However, this is hitherto the first case we know of describing PEH in a patient with IBD. Both MEDLINE and OVID searches for epithelioid hemangioendothelioma, Crohn's disease, ulcerative colitis, and inflammatory bowel disease yielded only one reference. No cases of PEH in the setting of IBD were identified. At least one previous case study has described hepatic epithelioid hemangioendothelioma in association with IBD [5]. Increased levels of vascular endothelial growth factor have been shown to be upregulated in patients with IBD, suggesting increased angiogenesis in the setting of IBD [5]. It may be that chronic increase in inflammatory cytokines prompting increased angiogenesis in the endothelium primes this site as a potential nidus for primary disease [5]. While there is currently insufficient data to fully explore this, further study may shed light on a possible association between chronic inflammatory conditions and this rare endothelial neoplasm.
